# Predominance of Metabolic Resistance in a Six-Way-Resistant Palmer Amaranth (*Amaranthus palmeri*) Population

**DOI:** 10.3389/fpls.2020.614618

**Published:** 2021-01-14

**Authors:** Chandrima Shyam, Ednaldo A. Borgato, Dallas E. Peterson, Johanna Anita Dille, Mithila Jugulam

**Affiliations:** Department of Agronomy, Kansas State University, Manhattan, KS, United States

**Keywords:** metabolism, inhibitor assays, *EPSPS* amplification, cytochrome P450 monooxygenases, glutathione *S*-transferases

## Abstract

Evolution of multiple herbicide resistance in Palmer amaranth across the United States is a serious challenge for its management. Recently, a Palmer amaranth population (KCTR; Kansas Conservation Tillage Resistant) from a long-term conservation tillage research project in Kansas, United States, was found uncontrolled by several commonly used herbicides. Importantly, this field did not have a history of repeated use of some of the herbicides for which the KCTR Palmer amaranth population showed lack of control. The objectives of this study were to confirm the evolution of multiple resistances and determine possible mechanism(s) of resistance in KCTR Palmer amaranth plants. In response to post-emergence application, 28–100% of KCTR Palmer amaranth survived field recommended rates of 2,4-D, ALS-, PS II-, EPSPS-, PPO-, HPPD-inhibitor herbicides, or tank- or pre-mixture of PS II- and HPPD-inhibitor herbicides, confirming evolution of six-way resistance in this Palmer amaranth population. However, this population was found susceptible to the PS I- and glutamine synthetase inhibitor herbicides. Chlorsulfuron-, imazethapyr-, and atrazine-resistant plants did not show any previously reported mutation in *ALS* and *psbA* genes, the target sites of these herbicides, respectively. However, the survivors of glyphosate treatment showed amplification of *EPSPS* gene (up to 88 copies). The KCTR plants pretreated with cytochrome P450 or GST inhibitors along with atrazine, 2,4-D, lactofen, or mesotrione had significantly less biomass accumulation than those treated with herbicides alone. Plants treated with P450 inhibitor followed by imazethapyr showed moderate reduction of biomass in KCTR which was statistically similar to a susceptible Palmer amaranth population treated with imazethapyr. These results suggest predominance of metabolic resistance possibly mediated by cytochrome P450 and GST enzyme activity that may have predisposed the KCTR Palmer amaranth population to evolve resistance to multiple herbicides. This is the first report of evolution of six-way resistance in a single Palmer amaranth population. Appropriate management strategies, including integration of cultural, and mechanical, and herbicide mixtures, are warranted to control such Palmer amaranth populations.

## Introduction

Palmer amaranth (*Amaranthus palmeri* S. Watson) is a top-ranked troublesome weed in the United States (Van Wychen, 2019). It is a summer annual C_4_ eudicot species, with a fast growth rate and ability to accumulate biomass (Horak and Loughin, 2000; Sellers et al., 2003). These biological attributes make Palmer amaranth a highly competitive species and if uncontrolled can contribute to drastic yield losses of up to 91% in corn, 79% in soybean, 59% in cotton, and 50% in sorghum (Massinga et al., 2001; Morgan et al., 2001; Bensch et al., 2003; Moore et al., 2004). Palmer amaranth has currently evolved resistance to eight herbicide sites of action (SOAs) in the United States, including 5-enolpyruvylshikimate-3-phosphate synthase (EPSPS-), acetolactate synthase- (ALS-), photosystem II- (PS II-), 4-hydroxyphenylpyruvate dioxygenase- (HPPD-), protoporphyrinogen oxidase- (PPO-), long-chain fatty acid- (LCFA-), microtubule-inhibitor herbicides, and synthetic auxins (Heap, 2020). Previously, resistance to up to five SOAs, i.e., ALS-, EPSPS-, PS II-, HPPD-inhibitor herbicides, and synthetic auxins, was reported in different populations of Palmer amaranth from Kansas (Nakka et al., 2017a, b, c; Kumar et al., 2019; Chaudhari et al., 2020).

Weed resistance to herbicides, especially multiple-herbicide resistance, poses a serious threat to global food production. Both target-site (TSR) and non-target-site (NTSR) resistance mechanisms have been found to confer resistance to herbicides in Palmer amaranth. TSR mechanisms involving alterations in the target site of the herbicide such as amino acid substitutions or deletions and increased copy number and/or expression of the target gene have been reported in this species (Gaines et al., 2010; Salas et al., 2016; Nakka et al., 2017b, c). Mutations in the gene encoding herbicide-targeted enzymes can reduce herbicide-binding activity leading to resistance. Specifically, in Palmer amaranth single amino acid substitutions, i.e., A122S or A122T, P197S or P197A, T574L, and S653A were reported to confer resistance to ALS-inhibitors in weed species (Küpper et al., 2017; Nakka et al., 2017c; Singh et al., 2019). Palmer amaranth populations resistant to PPO-inhibitor herbicides were found to have the amino acid substitutions R128M/G (also referred as R98), and G399A, as well as a codon (glycine) deletion at the position 210 (Δ210) in *PPX2* gene coding for the target enzyme of PPO-inhibitor herbicides (Salas et al., 2016; Giacomini et al., 2017; Salas-Perez et al., 2017; Varanasi et al., 2017; Rangani et al., 2019). Another commonly identified TSR mechanism in Palmer amaranth is the amplification of the *EPSPS* gene, the molecular target of glyphosate (Gaines et al., 2010; Chahal et al., 2017; Molin et al., 2017; Singh et al., 2018). Furthermore, the amplified *EPSPS* gene copies are present in the form of extrachromosomal circular DNA (eccDNA), with an autonomous replication site, and are randomly inherited during cell division (Koo et al., 2018; Molin et al., 2020). After the first case of glyphosate-resistant Palmer amaranth from Georgia, United States in 2005, it has rapidly spread throughout the United States becoming a serious challenge for agriculture. Apart from *EPSPS* amplification, a mutation in the *EPSPS* gene leading to P102S substitution has also been reported in Palmer amaranth (Kaundun et al., 2019).

In contrast to TSR, NTSR mechanisms do not directly alter the target site but reduce the amount of active herbicide reaching the target site due to either reduced absorption, translocation, or increased metabolism of the herbicide. Specifically, in metabolic resistance, the active herbicide is broken down into non-toxic forms before it reaches the target site, thus reducing its efficacy. Reduced absorption and translocation imparting glyphosate resistance in a Palmer amaranth population from Argentina have been reported (Palma-Bautista et al., 2019). However, enhanced herbicide detoxification *via* cytochrome P450 monooxygenase (P450s) and glutathione *S*-transferase (GSTs) activity is the most common NTSR mechanism reported in ALS-, PS II-, HPPD-, and PPO-inhibitor-resistant Palmer amaranth (Nakka et al., 2017a, b, c; Varanasi et al., 2018). Additionally, GSTs were found to be involved in *S*-metolachlor resistance in this species (Brabham et al., 2019).

P450s and GSTs are groups of enzymes important to the catalysis of several xenobiotic compounds in living organisms, including herbicides in many crops and weeds (Dixon et al., 2010; Pandian et al., 2020). Importantly, metabolic resistance can confer cross- or multiple-resistance (Jugulam and Shyam, 2019). The P450s from CYP81A subfamily have been shown to impart cross-resistance to several herbicide classes including ALS-, ACCase-, PS II-, phytoene desaturase- (PDS-), PPO-, HPPD-, and 1-deoxy-D-xylulose 5-phosphate synthase- (DOPX-) inhibitor herbicides in late watergrass (*Echinochloa phyllopogon*; Dimaano et al., 2020). Likewise, a phi (F) class GST, *AmGSTF1*, was shown to detoxify multiple herbicides in blackgrass (*Alopecurus myosuroides*; Cummins et al., 2013).

We previously reported a Palmer amaranth population from Kansas (KSR) with resistance to ALS-, PS II-, and HPPD-inhibitor herbicides (Nakka et al., 2017a, b, c). In this population, predominance of metabolic resistance *via* P450 or G*S*T activity to all the above herbicides was found (Nakka et al., 2017a, b, c). Importantly, the field where the KSR population was collected had a history of use of ALS- and PS II-, but not HPPD-inhibitor herbicides, validating the implications of metabolic resistance in bestowing cross-resistance to herbicides that were not previously used (Nakka et al., 2017b; Nandula et al., 2019). More recently, a three-way resistant Palmer amaranth population (HMR) was documented in Kansas with resistance to ALS-, PS II-, and EPSPS-inhibitor herbicides (Chaudhari et al., 2020). Several mutations in the *ALS* gene, such as P197S, P197T, P197A, and P197A, or T574L were documented in HMR. *EPSPS* amplification was observed in this population with copies ranging from 50 to 140. No mutation was observed in *psbA* gene fragments of this population implying the involvement of NTSR imparting atrazine resistance.

In 2018, a Palmer amaranth population (KCTR) from a long-term conservation tillage experimental field (Department of Agronomy, Kansas State University) grown with continuous sorghum for over 45 years was found to survive post-emergence (POST) application of several commonly used herbicides, including 2,4-D and atrazine. These herbicides have been routinely used in this field to control broadleaf weeds. Since Palmer amaranth has evolved resistance to eight SOAs (Heap, 2020) and there was a predominance of metabolic resistance in other Palmer amaranth populations in Kansas, the KCTR Palmer amaranth was used in this research to confirm and characterize resistance. The objectives of this research were to (1) confirm evolution of resistance in KCTR to several POST herbicides and (2) determine if TSR or NTSR mechanisms confer resistance to multiple herbicides.

## Materials and Methods

### Plant Material and Growing Conditions

Ten KCTR plants that survived 2,4-D treatment (560 g ae ha^–1^) and showed active growth after herbicide application were collected (summer 2018) and brought to the weed science greenhouse at Kansas State University. These plants were transplanted into pots (15 × 10 × 15 cm) for seed production. Seeds produced from several female plants were harvested, cleaned, and pooled to evaluate their response to multiple herbicides. Susceptible populations including one from Kansas (KSS) and one from Mississippi (MSS) were used for comparisons. All experiments were conducted in the above greenhouse maintained at 30/23°C ± 2°C (day/night temperatures) with 60% ± 10% relative humidity, and 14/10 h day/night photoperiod supplemented with 250 μmol m^–2^ s^–1^ illumination provided by sodium vapor lamps.

### Response of KCTR to Post-emergence (POST) Herbicide Application

Seeds of KCTR, KSS, and MSS populations were germinated in plastic trays (21 × 6 × 4 cm) filled with a commercial potting mixture (Pro-Mix^®^ premium potting mix, Premier Tech Home and Garden Inc., Ontario, CA). After emergence, seedlings were individually transplanted into pots (6 × 6 × 6.5 cm) and grown under greenhouse conditions as previously described. This experiment was performed in a completely randomized design with 18 treatments including field recommended rates of 17 POST herbicides (Table 1) and non-treated control. Twenty-five replicates were maintained for each treatment, and the experiment was repeated. In total, 50 plants (from two runs) from each of the KCTR and KSS or MSS population were treated with these POST herbicides (Table 1). Adjuvants were included following manufacturer instructions (Table 1). Herbicides were applied using a bench-track sprayer (Generation III, DeVries Manufacturing, RR1 Box 184, Hollandale, MN) equipped with a flat-fan nozzle tip (8002 Teejet, Spraying Systems Co., Wheaton, IL) calibrated to deliver a spray volume proportional to 187 L ha^–1^ at 4.77 km h^–1^. Plant survival (alive or dead) was assessed at 2 weeks after treatment (WAT) with PPO-inhibitor herbicides; 3 WAT for glyphosate, ALS-, HPPD-, and PS II-inhibitor herbicides; and 4 WAT for 2,4-D. The percent survival (Table 2) was calculated by dividing the number of plants that survived herbicide by total number of plants treated, considering both experimental runs.

**TABLE 1:** POST-emergence herbicide treatments used for screening KCTR, MSS, and KSS Palmer amaranth populations.

WSSA group	SOA	Herbicides	Dose^a^ (g ha^–1^)	Product	Manufacturer
2	ALS inhibitors	Chlorsulfuron	18	Glean^® b^	Corteva Agriscience, Willington, DE
		Thifensulfuron	36	Harmony SG^b^	Corteva Agriscience
		Imazamox	35	Beyond^b^	BASF Corp., Research Triangle Park, NC
		Imazethapyr	36	Pursuit^b^	BASF Corp.
4	Synthetic auxins	2,4-D	560	2,4-D 4L Amine	Winfield Solutions, LLC, St. Paul, MN
5	PS II inhibitors	Atrazine	2,240	Aatrex 4L^c^	Syngenta Crop Protection, LLC., Greensboro, NC
		Metribuzin	140	Sencor 75^c^	Bayer Crop Science, Centreway Green Way, NC
9	EPSPS inhibitors	Glyphosate	840	Roundup WeatherMAX^d^	Bayer Crop Science
10	Glutamine synthetase inhibitors	Glufosinate	655	Liberty 280 SL^d^	BASF Corporation
14	PPO inhibitors	Lactofen	175	Cobra^c^	Valent U.S.A. Corp., Walnut Creek, CA
		Fomesafen	264	Flexstar^b^	Syngenta Crop Protection
22	PS I inhibitors	Paraquat	560	Gramaxone SL 2.0^c^	Syngenta Crop Protection
27	HPPD inhibitors	Mesotrione	105	Callisto^c^	Syngenta Crop Protection
		Tembotrione	92	Laudis^de^	Bayer Crop Science
5 + 27	PS II + HPPD inhibitors	Atrazine + mesotrione	1,120 + 105	Aatrex 4L + Calisto^c^	Syngenta Crop Protection
		Bromoxynil + pyrasulfotole	288	Huskie^b,d^	Bayer Crop Science

*^a^Field recommended rate to control Palmer amaranth. ^b^Nonionic surfactant at 0.25% v/v. ^c^Crop oil concentrate at 1% v/v. ^d^Ammonium sulfate (34%) at 2% v/v; methylated seed oil at 1% v/v. ^e^Methylated seed oil at 1% v/v.*

**TABLE 2:** Percent survival of KCTR Palmer amaranth population to different post-emergence herbicides.

WSSA group	SOA	Herbicides^a^	Survival^b^ (%)
2	ALS inhibitors	Chlorsulfuron	34
		Thifensulfuron	60
		Imazamox	70
		Imazethapyr	60
4	Synthetic auxins	2,4-D	84
5	PS II inhibitors	Atrazine	100
		Metribuzin	36
9	EPSPS inhibitors	Glyphosate	28
10	Glutamine synthetase inhibitors	Glufosinate	0
14	PPO inhibitors	Lactofen	84
		Fomesafen	29
22	PS I inhibitors	Paraquat	0
27	HPPD inhibitors	Mesotrione	90
		Tembotrione	84
5 + 27	PS II inhibitors + HPPD inhibitors	Atrazine + mesotrione	42
		Bromoxynil + pyrasulfotole	98

*^a^A total of 50 plants from each population (KCTR and KSS or MSS) were sprayed with each herbicide. ^b^KSS and MSS were controlled (≥ 95%) with all the herbicides.*

### Assessment of TSR Mechanisms in KCTR Palmer Amaranth

#### DNA Isolation and Sequence Comparisons of *ALS* and *psbA* Genes in KCTR, KSS, and MSS Palmer Amaranth

Approximately 100 mg of young leaf tissue was collected from the survivors of chlorsulfuron- (*n* = 3; n: number of plants) and imazethapyr- (*n* = 16) and atrazine (*n* = 22) treated KCTR, and non-treated KSS (*n* = 1) and MSS (*n* = 5) plants for DNA isolation. After collection, leaf tissue was homogenized using a prechilled mortar and pestle with liquid nitrogen. Total genomic DNA (gDNA) was extracted using a Genomic DNA Extraction kit (Thermo Fisher Scientific, Waltham, MA). DNA was quantified using a nanodrop (Nanodrop 1000, Thermo Fisher Scientific), and quality was verified using 0.8% agarose gel electrophoresis prior to further steps. Polymerase chain reactions (PCR) were performed using T100^TM^ Thermal Cycler (Bio-Rad Inc., Hercules, CA) to amplify the *ALS* and *psbA* genes, the target site of these herbicides. Individual reactions included 80 ng of DNA, 2 μL of each forward and reverse primers (5 μM), 10 μL of PCR master mix (GoTaq Green Master Mix, 2×, Promega PCR Master Mix, Fisher Scientific Company, Ontario, Canada), and molecular-grade water totalizing 25 μL per reaction. Primer sets used to amplify the *ALS* and *psbA* genes were designed by Mengistu et al. (2005) and Whaley et al. (2007), respectively, and have previously been used in our lab (Nakka et al., 2017a, c). For *ALS* gene amplification, the following PCR conditions were maintained: 95°C for 5 min and 35 × 95°C for 1 min, 57°C for 30 s, 2 min at 72°C, and 10 min at 72°C. PCR conditions consisted of 95°C for 5 min for initial denaturation and 35 cycles of 95°C for 30 s for denaturation, 55°C for 30 s for annealing, 72°C for 45 s for extension, and 10 min at 72°C for final extension for *psbA*. PCR products were purified using GeneJET PCR Purification Kit (Thermo Fisher Scientific) following the manufacturer instructions and sent for Sanger sequencing at the Genewiz facilities (Genewiz Inc., South Plainfield, NJ). For *ALS* sequencing, along with the forward and reverse primers used for PCR, an internal primer (ALS_F2-5′-AACAGCCCATTAAATTGGGTG-3′) was used. The *psbA* gene was sequenced with the same forward primer used for PCR. Multiple alignments of *ALS* and *psbA* gene sequences of KCTR, KSS, and MSS sequences were performed using Geneious Prime^®^ software (Biomatters Inc., Newark, NJ).

#### Relative *EPSPS* Genomic Copy Number Estimation

Leaf tissue of KCTR plants (*n* = 13) that survived glyphosate treatment were collected to estimate the *EPSPS* copy number relative to β-*Tubulin* using a real-time quantitative PCR (RT-qPCR). DNA extraction was performed as described above, and qPCR was performed using a StepOnePlus^TM^ real-time detection system (Applied Biosystems, Waltham, MA). Each qPCR reaction consisted of 8 μL of PowerUp^TM^ SYBR^TM^ Green master mix (Applied Biosystems), 2 μL each of forward and reverse primers (5 μM) (Gaines et al., 2010), and 2 μL of gDNA (20 ng μL^–1^) with 14 μL total. *β-Tubulin* was used as endogenous control as described by Godar et al. (2015). Individual reactions were performed with DNA collected from thirteen different KCTR plants that survived glyphosate application as biological replicates, with three technical replicates per DNA sample for both target and endogenous control genes. This experiment was repeated, and data was combined. To determine the specificity of the qPCR products, a melt curve profile was included following the thermal cycling. The *EPSPS* copy number in KCTR was estimated using the formula for fold induction (2^–ΔΔCt^) (Pfaffl, 2001) relative to the reference sample, i.e., glyphosate-susceptible KSS or MSS plants, in each run. The mean *EPSPS* copy number of KCTR plants along with susceptible KSS and MSS were plotted along with standard error of mean calculated from two experimental runs.

### POST Herbicide Efficacy With Cytochrome P450 and GST Inhibitors for Assessing Presence of NTSR Mechanisms in KCTR Palmer Amaranth

Whole-plant bioassays were conducted to investigate the presence of P450- and GST-mediated metabolic resistance to the herbicides (imazethapyr, atrazine, 2,4-D, mesotrione, and lactofen) for which Palmer amaranth and common waterhemp (*Amaranthus tuberculatus*) has been reported to have evolved such resistance. This experiment was performed under a completely randomized design with a factorial arrangement and was repeated. Treatments included combination of Palmer amaranth populations (resistant and susceptible) and chemical treatments (described below), with at least 8 replicates. Resistant (KCTR) and susceptible (KSS or MSS; based on availability of seeds) Palmer amaranth populations were compared. Chemical treatments included a) herbicide only, b) enzymatic inhibitor only (either P450 and/or GST inhibitor; depending on the herbicide), c) combination of enzymatic inhibitor with a herbicide, and d) a non-treated control. Herbicide doses included application of field recommended rates of imazethapyr, mesotrione, atrazine, lactofen, and 2,4-D (Table 1). Based on published literature, both P450 and GST inhibitors were included to evaluate the metabolic resistance to lactofen for a total of six treatments, i.e., (a) lactofen only, (b) malathion only, (c) NBD-Cl only, (d) combination of malathion with lactofen, (e) combination of NBD-Cl with lactofen, and (f) non-treated control. The treatments were applied using a bench-track sprayer with appropriate adjuvants as described before. Malathion (Spectracide^®^, Spectrum Group, St. Louis, MO), a P450 inhibitor, was applied at 2,000 g ai ha^–1^ at least 30 min prior to herbicide application and also soil-applied (5 mM, 50 mL solution pot^–1^) at 48 h after herbicide application as described by Ma et al. (2013) in combinations with or without imazethapyr, lactofen, and mesotrione. Our preliminary study (unpublished) to test the effect of malathion at 1,500 and 2,000 g ai ha^–1^ on 2,4-D efficacy in 2,4-D-resistant common waterhemp from Nebraska indicated that malathion at 1,500 g ai ha^–1^ was sufficient to increase susceptibility of common waterhemp to 2,4-D. Palmer amaranth is a close relative of common waterhemp; therefore, for assessing 2,4-D metabolism in KCTR, malathion was used at 1,500 g ai ha^–1^, followed by soil application as described above. The GST inhibitor, NBD-Cl (Sigma Aldrich, St. Louis, MO), was applied at 270 g ai ha^–1^, 48 h before atrazine or lactofen applications (Ma et al., 2013). Experiments were performed twice. Aboveground biomass was harvested at 2 WAT for lactofen; 3 WAT for glyphosate, imazethapyr, mesotrione, and atrazine; and 4 WAT for 2,4-D, oven-dried at 65°C for 72 h, and quantified. Biomass data were converted to percent dry weight relative to the non-treated control for statistical analysis.

Levene’s test (α = 0.05) was conducted to compare runs, and if no significance was identified, relative dry weight data were combined. Normality of residuals and homoscedasticity of variances were verified prior to ANOVA, and relative dry weight data was square root-transformed. Data were subsequently fitted to a linear mixed effect model using the “*nlme*” package and the function “*lme*” available in R (version 4.0.3, R Core Team, 2020) with the R-Studio 9.4 interface (R Studio, PBC, Boston, MA) considering Palmer amaranth populations and chemical treatments as fixed effect and experimental runs as a random effect. If interaction across populations and treatments was significant, the means were separated using Tukey’s test using the “*multcompview*” and “*lsmeans*” packages at α = 0.05. Data for adjusted means were back-transformed to calculate percent reduction in biomass with application of inhibitors in comparison to herbicide alone. These results were plotted using “*ggplot2*” package for graphical visualizations.

## Results

### Percent Survival of KCTR and KSS or MSS in Response to POST Herbicide Treatment

**FIGURE 1:**
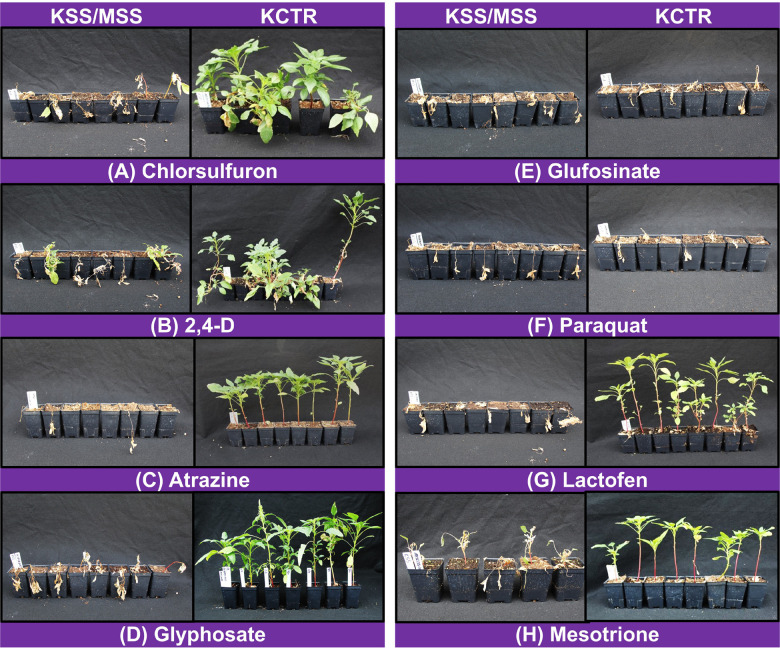
Response of susceptible (MSS or KSS) and resistant (KCTR) Palmer amaranth to chlorsulfuron **(A)**, 2,4-D **(B)**, atrazine **(C)**, glyphosate **(D)**, glufosinate **(E)**, paraquat **(F)**, lactofen **(G)**, and mesotrione **(H)** application 2–4 weeks after treatment.

Percent survival of KCTR plants to different herbicides was highly variable indicating the considerable genetic variability KCTR Palmer amaranth population. Overall, >28% of KCTR plants survived field-recommended rates of all herbicides tested, except paraquat and glufosinate, for which this population was found to be susceptible (Table 2). Following 2,4-D treatment, KSS plants (∼10%) were recorded with green tissue, weak twisted stem, and dried meristem (Figure 1). However, since no growth as well as presence of dried meristem was observed following 2,4-D treatment, these plants were considered as dead. Overall, > 95% control of either KSS or MSS plants was recorded with all herbicide treatments (Table 2). The lowest percent survival of KCTR plants was found for glyphosate (28%). In response to several ALS-inhibitor herbicides, the KCTR plants showed variation in % survival as follows: 34% for chlorsulfuron, 60% for thifensulfuron and imazethapyr, and 70% for imazamox suggesting that KCTR has evolved resistance to both sulfonylureas and imidazolines.

In response to PS II-inhibitor (e.g., atrazine and metribuzin) application, all KCTR plants survived atrazine but only 36% survived treatment with metribuzin, confirming the evolution of resistance to PS II-inhibitor herbicides (Table 2). Eighty-four and 90% of KCTR plants survived mesotrione and tembotrione treatments, respectively, suggesting prevalence of resistance to HPPD-inhibitor herbicides (Table 2). Nonetheless, 42% of KCTR plants survived the tank mixture of atrazine and mesotrione (Table 2). Additionally, 98% of KCTR plants also survived the commercial premix of bromoxynil (PS II-inhibitor) + pyrasulfotole (HPPD-inhibitor), one of the widely used POST herbicides for Palmer amaranth control in grain sorghum production. In response to PPO-inhibitor applications, 29 and 84% of KCTR plants survived treatment with fomesafen and lactofen, respectively, confirming evolved resistance to PPO-inhibitor herbicides (Table 2). Also, 84% of KCTR plants survived 2,4-D treatment at the field recommended rate.

### Assessment of TSR Mechanisms in KCTR Palmer Amaranth

**FIGURE 2:**
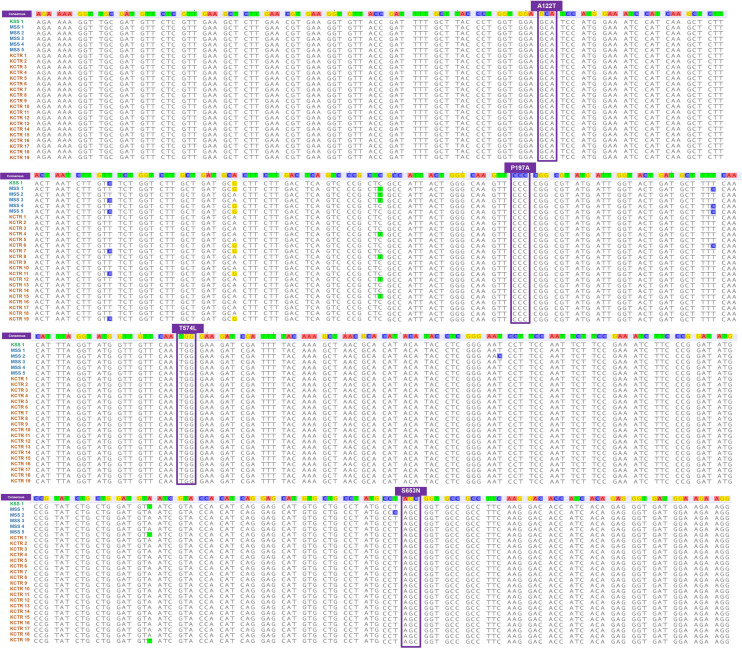
Nucleotide sequence alignment of *ALS* gene fragment from susceptible (MSS and KSS) and chlorsulfuron (KCTR 1–3)- and imazethapyr (KCTR 4–19)-resistant Palmer amaranth. Nucleotide polymorphisms were observed across KCTR, KSS, and MSS Palmer amaranth, but no amino acid substitutions were found.

**FIGURE 3:**
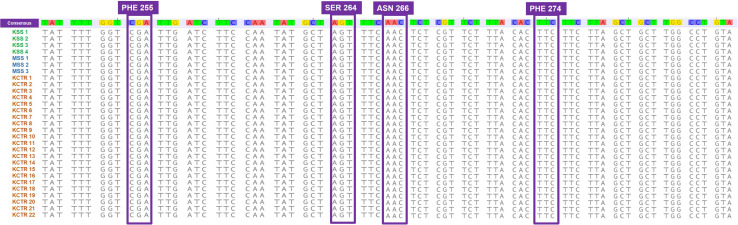
Nucleotide sequence alignment of *psbA* gene fragments from atrazine-susceptible (MSS and KSS) and -resistant (KCTR) Palmer amaranth individuals. No nucleotide polymorphisms were observed among KCTR, KSS, and MSS Palmer amaranth individuals.

**FIGURE 4:**
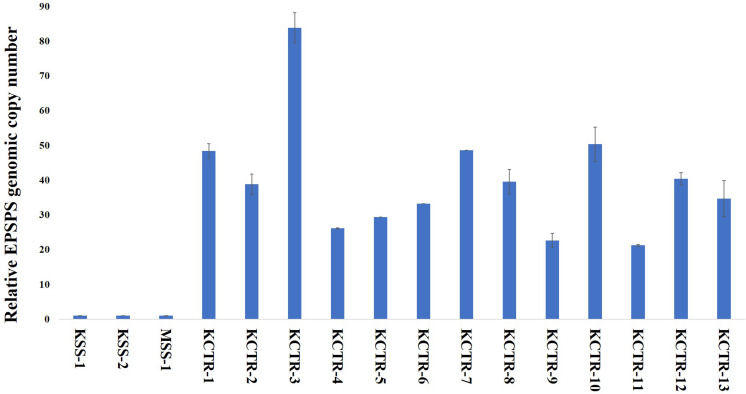
*EPSPS* genomic copy number in glyphosate-susceptible (MSS and KSS) and glyphosate-resistant (KCTR) Palmer amaranth plants relative to the susceptible plants. KCTR plants were treated with the recommended field rate of glyphosate. Error bars represent the standard error from the mean (2 runs and in each run 3 technical replicates). The qPCR data was normalized using β-tubulin as the reference gene.

Nucleotide sequence alignment of the *ALS* gene of the KCTR, KSS, and MSS plants showed lack of the four previously reported mutations (Nakka et al., 2017c; Küpper et al., 2017; Singh et al., 2019; Chaudhari et al., 2020) known to confer resistance ALS-inhibitor herbicides in Palmer amaranth (Figure 2). Even though some nucleotide polymorphisms were detected, none of them were consistent among the resistant plants or resulted in amino acid substitution (Figure 2). No nucleotide polymorphisms were detected in the *psbA* sequence of KCTR plants (Figure 3). Our qPCR results indicated that glyphosate-resistant KCTR plants had increased number of *EPSPS* copies, ranging from 20 to 88, compared to KSS or MSS (Figure 4).

### Assessment of NTSR Mechanisms in KCTR Palmer Amaranth

**FIGURE 5:**
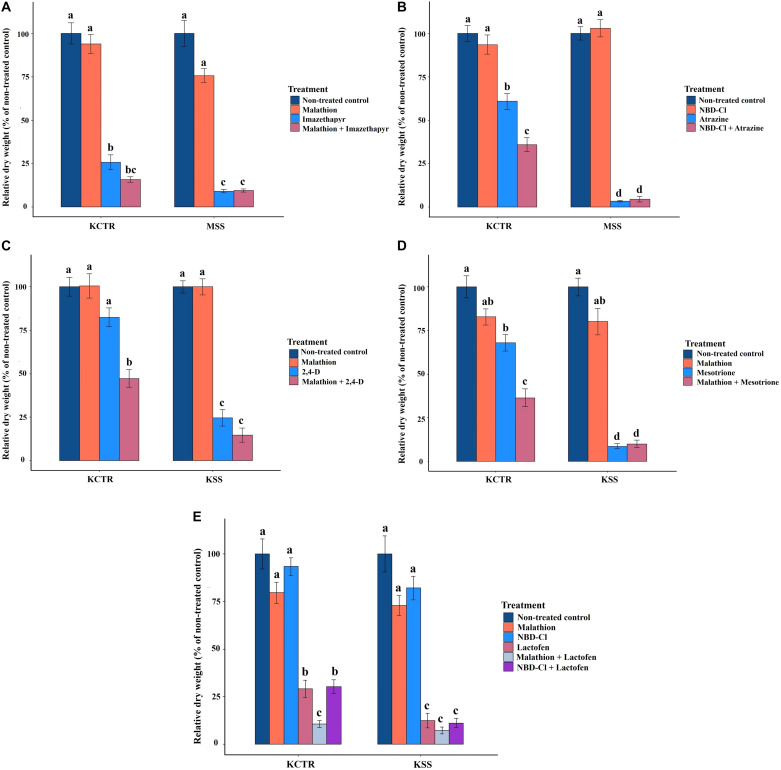
Effect of P450 and GST inhibitors on efficacy of imazethapyr **(A)**, atrazine **(B)**, 2,4-D **(C)**, mesotrione **(D)**, and lactofen **(E)** in susceptible (KSS or MSS) and resistant (KCTR) Palmer amaranth populations. Error bars represent standard error of the mean. Letters represent significant differences identified by separation of means using Tukey (5%).

Malathion treatment alone did not significantly impact biomass accumulation in either KCTR, KSS or MSS plants (Figures 5A,C–E). Contrary to that, imazethapyr treatment resulted in significantly (*p* < 0.0001) lower biomass accumulation in MSS plants (9%) compared to KCTR plants (23%) (Figure 5A). Treatment of malathion with imazethapyr did not reduce the relative biomass accumulation of KCTR plants compared to KCTR plants treated with imazethapyr alone (Figure 5A). Interestingly, there was no significant difference between KCTR plants treated with malathion with imazethapyr (15%) in comparison to MSS plants treated with either imazethapyr alone (9%) or malathion with imazethapyr (8%; Figure 5A).

Similar to malathion, NBD-Cl treatment alone did not significantly affect biomass accumulation in either KCTR, KSS, or MSS plants (Figures 5B,E). Atrazine treatment at the field-recommended rate resulted in 58% relative biomass accumulation in KCTR, whereas MSS plants had 3% biomass accumulation (Figure 5B). This is similar to the results of the herbicide screening experiment (Table 2). Treatment of NBD-Cl 48 h prior to atrazine resulted in only 33% relative biomass accumulation in KCTR plants which was significantly lower than atrazine only treatment (58%; *p* < 0.0001). Such effect of malathion was not observed with pretreatment of NBD-Cl in MSS plants (Figure 5B).

Highly variable biomass accumulation was observed in KCTR plants treated with 2,4-D; however, relative biomass accumulation in 2,4-D-treated KCTR plants (81%) was higher than 2,4-D-treated KSS plants (21%) (Figure 5C). Treatment of malathion with 2,4-D resulted in statistically lower biomass accumulation (45%) than only 2,4-D-treated KCTR plants (*p* = 0.0005). However, such impact of malathion was not observed in KSS plants (Figure 5C).

Mesotrione treatment resulted in significantly lower biomass accumulation in KCTR plants (65%) compared to KSS plants (8%) (Figure 5D). Nonetheless, treatment of malathion with mesotrione resulted in lower biomass accumulation in KCTR plants (31%) than mesotrione treatment alone (*p* < 0.0001) (Figure 5D). Contrary to that, malathion- and mesotrione-treated KSS plants (8%) showed no difference in biomass accumulation compared to mesotrione only treatment (Figure 5D).

Lactofen-only treatment resulted in significantly lower (*p* = 0.0418) relative biomass accumulation in KCTR plants (26%) compared to KSS plants (11%). Even with significant biomass reduction in the resistant population, several plants survived this herbicide application (Table 2 and Figure 5E). The KSS Palmer amaranth was susceptible to all treatments containing lactofen (Table 2 and Figure 5E). Treatment of NBD-Cl fb lactofen did not result in increased sensitivity of KCTR (Figure 5E). However, combination of malathion with lactofen significantly reduced biomass accumulation in KCTR plants (8%), compared to the lactofen-only treatment. Such impact of malathion was not observed in KSS plants (Figure 5E).

## Discussion

Palmer amaranth is a dioecious species with prolific seed production that has already evolved resistance to eight herbicide SOAs (Heap, 2020). In this research, we report for the first time the evolution of six-way resistance in a single Palmer amaranth population, i.e., KCTR with predominance of metabolic resistance mechanisms. Previously Palmer amaranth populations with resistance to three and five SOAs have been reported (Nakka et al., 2017a, b, c; Kumar et al., 2019; Chaudhari et al., 2020). Similar to our findings, the six-way resistant common waterhemp population was also documented in Missouri (Shergill et al., 2018). Our results confirm the evolution of resistance to ALS-, PS II-, HPPD-, PPO-, EPSPS-inhibitor herbicides, and synthetic auxins in the KCTR population (Table 2). KCTR plants survived (28–100%) application of these herbicides, while satisfactory control (0% survival) was achieved only with PS I- (e.g., paraquat) and glutamine synthetase inhibitor herbicides (e.g., glufosinate) (Table 2). Such a wide range of survival for different SOAs indicates that KCTR is likely to be not genetically homogeneous, and possibly there is ongoing segregation for resistance to these SOAs, especially ALS- and EPSPS-inhibitor herbicides (Table 2). This is not unusual considering the amount of genetic variability offered by an outcrossing weed species like Palmer amaranth. Improved control of KCTR plants (58%) was observed with a tank mixture of atrazine (half of the recommended rate: 1,120 g ai ha^–1^) and mesotrione (recommended rate: 105 g ai ha^–1^) (Table 1) compared to atrazine alone (0%) or mesotrione alone (16%). However, some plants were not effectively controlled by this tank mixture. Such improvement in control can be attributed to the synergism often observed with tank mixing PS II- and HPPD-inhibitor herbicides (Abendroth et al., 2006; Chahal et al., 2019a). Additionally, the commercial pre-mixture of bromoxynil (PS II-inhibitor) and pyrasulfotole (HPPD-inhibitor) performed poorly in comparison to a tank mix of atrazine and mesotrione.

Although mutations in herbicide target genes conferring resistance are rare evolutionary events, mutations in the *ALS* gene at eight positions (four positions in Palmer amaranth) were found to confer resistance to several classes of ALS inhibitors in weeds (Tranel and Wright, 2002; Yu and Powles, 2014; Heap, 2020). Several amino acid substitutions at the *ALS* gene have been documented in Palmer amaranth populations from Kansas (Nakka et al., 2017c; Chaudhari et al., 2020). Interestingly, upon sequencing the whole *ALS* gene, the KCTR plants showed no presence of any of these substitutions (Figure 2). Malathion has been shown to increase the metabolic half-life of herbicides by inhibiting P450-dependent metabolism (Kreuz and Fonné-Pfister, 1992). Therefore, malathion treatment was done prior and after imazethapyr to test involvement of P450-based detoxification mechanisms in KCTR. Even though imazethapyr treatment was not significantly different from the combination of malathion with imazethapyr in reducing KCTR biomass, malathion with imazethapyr-treated KCTR plants produced the same level of biomass as imazethapyr-treated MSS plants (Figure 5A). This indicated moderate reduction of biomass in KCTR with malathion treatment. Moreover, since only 60% of KCTR plants were found to be resistant to imazethapyr (Table 2), it is possible that sensitive plants in KCTR may have contributed to the lack of differences between imazethapyr only and combination of malathion with imazethapyr treatment. Different P450 inhibitors have been observed to have varying levels of synergistic effect in resistant weeds depending on the herbicide as well as P450 isozyme. For instance, Preston et al. (1996) observed piperonyl butoxide (PBO; another P450 inhibitor) inhibiting chlorotoluron and simazine resistance in rigid ryegrass (*Lolium rigidum*), but malathion failed to do so. Similarly, Oliveira et al. (2018) observed that malathion improved the efficacy of tembotrione but not of mesotrione in HPPD-inhibitor-resistant common waterhemp from Nebraska. Therefore, it is possible that different P450s, which are not completely inhibited by malathion, are involved in imparting resistance to imazethapyr in KCTR.

All KCTR plants survived atrazine application (Table 2), and the lack of any known mutations in the *psbA* suggests a NTSR mechanism to atrazine as reported in other Palmer amaranth populations (Nakka et al., 2017b; Chaudhari et al., 2020). A V219L mutation in the *psbA* gene was found in atrazine-resistant Powell amaranth (*Amaranthus powellii*; Dumont et al., 2016) but not in common waterhemp or other *Amaranthus* species closely related to Palmer amaranth (Ma et al., 2013; Shergill et al., 2018; Chahal et al., 2019b). NBD-Cl derivatives have been shown to give strong GST inhibition in human tumor cells and are termed as suicide inhibitors of GSTs (Ricci et al., 2005). Therefore, NBD-Cl treatment prior to atrazine application was given to KCTR plants to determine the involvement of GSTs in imparting resistance. This treatment significantly reduced biomass accumulation in KCTR plants indicating the involvement of GST enzymes in metabolizing atrazine. Previously, pretreatment with NBD-Cl has reversed atrazine resistance in common waterhemp (Ma et al., 2013; Shergill et al., 2018). Metabolic resistance to atrazine *via* glutathione conjugation mediated by GST activity has been reported in several Palmer amaranth and common waterhemp populations in the United States Midwest (Ma et al., 2013; Shergill et al., 2018; Chahal et al., 2019b). KCTR plants are also resistant to metribuzin, another PS II inhibitor. Metribuzin resistance mediated by enhanced metabolism was reported in wild radish (*Raphanus raphanistrum*; Lu et al., 2019) and rigid ryegrass (Ma et al., 2020).

Treatment of malathion with application of 2,4-D, mesotrione, or lactofen significantly reduced biomass accumulation in KCTR plants (Figures 5C,E), suggesting P450 enzymes-mediated detoxification of these herbicides in KCTR plants. Previously, malathion-induced reversal of weed resistance to 2,4-D (Shergill et al., 2018), carfentrazone, and fomesafen (Varanasi et al., 2018; Obenland et al., 2019), and mesotrione (Ma et al., 2013) has been reported. However, NBD-Cl failed to impact biomass accumulation in KCTR when applied prior to lactofen treatment. This indicates that certain P450 enzymes and not GSTs, or potentially specific GSTs not inhibited by NBD-Cl, may be involved in imparting resistance to lactofen in KCTR plants. In contrast to our findings, in the PPO-inhibitor-resistant Palmer amaranth population from Arkansas, the use of NBD-Cl caused the reversal of resistance to fomesafen (Varanasi et al., 2018).

*EPSPS* amplification (up to 88 copies) was found to contribute to glyphosate resistance in KCTR plants. Gaines et al. (2011) have shown that > 30 *EPSPS* copies are needed to withstand the field rate of glyphosate application (840 g ai ha^–1^) in Palmer amaranth. Despite a low percentage (28%; Table 2) of glyphosate survivors in this population, lack of fitness penalty associated with this resistance mechanism and the obligate outcrossing nature of Palmer amaranth can lead to the rapid spread and transfer to other susceptible populations *via* pollen (Sosnoskie et al., 2012; Giacomini et al., 2014).

Based on previous research in our laboratory, the coexistence of both TSR and NTSR for the same herbicide can occur in a single population or individual plant of Palmer amaranth (Nakka et al., 2017a, b; Chaudhari et al., 2020). Research is in progress to determine if such a scenario is present in KCTR as well. Future investigations focused on identifying specific P450s and GSTs involved in herbicide detoxification in this population will also be investigated. It is important to understand what predisposes a population to develop metabolic resistance to several SOAs, including those with no history of use and absence of selection pressure.

Because pre-emergence (PRE) herbicide treatments are regarded as one of the best strategies to manage herbicide resistance in weeds (Norsworthy et al., 2012), information on response of KCTR to PRE herbicides can help in formulating viable options to manage this population. Therefore, experiments are in progress to investigate the response of KCTR to several soil-applied residual PRE herbicides (e.g., atrazine, mesotrione, flumioxazin, and *S*-metolachlor) commonly used to control germinating and emerging seedlings of Palmer amaranth. Previously, there has been a fitness penalty associated with multiple-herbicide resistance in weed species such as rigid ryegrass (Vila-Aiub et al., 2005). Studies will also be conducted to assess whether any fitness penalty is present in this population as a result of evolution of six-way herbicide resistance, which can help in understanding the spread of resistance traits to other populations.

Evolution of resistance to six herbicide SOAs in the KCTR population leaves very few POST herbicide options for its control. Moreover, such accumulation of resistance traits in a single Palmer amaranth population poses serious questions on the effectiveness of stacked resistance traits in crops, such as 2,4-D + glyphosate + glufosinate or dicamba + glyphosate resistance in corn and beans. Considering the lack of introduction of new SOAs, it is crucial to conserve currently available SOAs to effectively manage weeds. Growers should be encouraged to adopt integrated weed management techniques to reduce selection pressure by herbicides and discourage further selection of the evolution of multiple resistance.

## Data Availability Statement

The authors acknowledge that the data presented in this study must be deposited and made publicly available in an acceptable repository, prior to publication (the accession number for ALS and psbA sequences are MW361337 and MW361342).

## Author Contributions

DP identified KCTR Palmer amaranth population in the conservation tillage field. MJ and DP conceived research hypothesis, methodology, led, and supervised the research. CS and EB conducted the research, collected, and analyzed data (equal contribution). JD critically reviewed the manuscript and provided valuable comments. All authors read, edited, and approved the final version of the manuscript.

## Conflict of Interest

The authors declare that the research was conducted in the absence of any commercial or financial relationships that could be construed as a potential conflict of interest.

## References

[B1] Abendroth J. A. Martin A. R. Roeth F. W. (2006). Plant response to combinations of mesotrione and photosystem II inhibitors. *Weed Technol.* 20 267–274. 10.1614/WT-05-020R.1

[B2] Bensch C. N. Horak M. J. Peterson D. E. (2003). Interference of redroot pigweed (*Amaranthus retroflexus*), Palmer amaranth (*A. palmeri*), and common waterhemp (*A. rudis*) in soybean. *Weed Sci.* 51 37–43. 10.1614/0043-1745(2003)051[0037:iorpar]2.0.co;2

[B3] Brabham C. Norsworthy J. K. Houston M. M. Varanasi V. K. Barber T. (2019). Confirmation of s-metolachlor resistance in Palmer amaranth (*Amaranthus palmeri*). *Weed Technol.* 33 720–726. 10.1017/wet.2019.44

[B4] Chahal P. S. Jugulam M. Jhala A. J. (2019a). Basis of atrazine and mesotrione synergism for controlling atrazine- and HPPD inhibitor-resistant Palmer amaranth. *Agron. J.* 111 3265–3273. 10.2134/agronj2019.01.0037

[B5] Chahal P. S. Jugulam M. Jhala A. J. (2019b). Mechanism of atrazine resistance in atrazine- and HPPD inhibitor-resistant Palmer amaranth (*Amaranthus palmeri* S. Watson) from Nebraska. *Can. J. Plant Sci.* 99 815–823. 10.1139/cjps-2018-0268

[B6] Chahal P. S. Varanasi V. K. Jugulam M. Jhala A. J. (2017). Glyphosate-resistant Palmer amaranth (*Amaranthus palmeri*) in Nebraska: confirmation, EPSPS gene amplification, and response to POST corn and soybean herbicides. *Weed Technol.* 31 80–93. 10.1614/WT-D-16-00109.1

[B7] Chaudhari S. Varanasi V. K. Nakka S. Bhowmik P. C. Thompson C. R. Peterson D. E. (2020). Evolution of target and non-target based multiple herbicide resistance in a single Palmer amaranth (*Amaranthus palmeri*) population from Kansas. *Weed Technol.* 34 447–453. 10.1017/wet.2020.32

[B8] Cummins I. Wortley D. J. Sabbadin F. He Z. Coxon C. R. Straker H. E. (2013). Key role for a glutathione transferase in multiple-herbicide resistance in grass weeds. *PNAS* 110 5812–5817. 10.1073/pnas.1221179110 23530204 PMC3625300

[B9] Dimaano N. G. Yamaguchi T. Fukunishi K. Tominaga T. Iwakami S. (2020). Functional characterization of cytochrome P450 CYP81A subfamily to disclose the pattern of cross-resistance in *Echinochloa phyllopogon*. *Plant Mol. Biol.* 102 403–416. 10.1007/s11103-019-00954-3 31898147

[B10] Dixon D. P. Skipsey M. Edwards R. (2010). Roles for glutathione transferases in plant secondary metabolism. *Phytochem* 71 338–350. 10.1016/j.phytochem.2009.12.012 20079507

[B11] Dumont M. Letarte J. Tardif F. J. (2016). Identification of a *psbA* mutation (Valine219 to Isoleucine) in powell amaranth (*Amaranthus powellii*) conferring resistance to linuron. *Weed Sci.* 64 6–11. 10.1614/WS-D-15-00087.1

[B12] Gaines T. A. Shaner D. L. Ward S. M. Leach J. E. Preston C. Westra P. (2011). Mechanism of resistance of evolved glyphosate-resistant Palmer amaranth (*Amaranthus palmeri*). *J. Agric. Food Chem.* 59 5886–5889. 10.1021/jf104719k 21329355

[B13] Gaines T. A. Zhang W. Wang D. Bukun B. Chisholm S. T. Shaner D. L. (2010). Gene amplification confers glyphosate resistance in *Amaranthus palmeri*. *PNAS* 107 1029–1034. 10.1073/pnas.0906649107 20018685 PMC2824275

[B14] Giacomini D. A. Umphres A. M. Nie H. Haozhen N. Mueller T. C. Steckel L. E. (2017). Two new PPX2 mutations associated with resistance to PPO-inhibiting herbicides in *Amaranthus palmeri*. *Pest Manag Sci.* 73 1559–1563. 10.1002/ps.4581 28370968

[B15] Giacomini D. A. Westra P. Ward S. M. (2014). Impact of genetic background in fitness cost studies: An example from glyphosate-resistant Palmer amaranth. *Weed Sci.* 62 29–37. 10.1614/WS-D-13-00066.1

[B16] Godar A. S. Stahlman P. W. Jugulam M. Dille J. A. (2015). Glyphosate-resistant Kochia in Kansas: *EPSPS* gene copy number in relation to resistance levels. *Weed Sci.* 63 587–595. 10.1614/WS-D-14-00145.1

[B17] Heap I. (2020). *The international survey of herbicide resistant weeds.* Available Online at: http://www.weedscience.org [Accessed September 08, 2020]

[B18] Horak M. J. Loughin T. M. (2000). Growth analysis of four *Amaranthus* species. *Weed Sci.* 48 347–355.

[B19] Jugulam M. Shyam C. (2019). Non-target-site resistance to herbicides: recent developments. *Plants* 8 417. 10.3390/plants8100417 31618956 PMC6843234

[B20] Kaundun S. S. Jackson L. V. Hutchings S. J. Galloway J. Marchegiani E. Howell A. (2019). Evolution of target-site resistance to glyphosate in an *Amaranthus palmeri* population from Argentina and its expression at different plant growth temperatures. *Plants* 8:512. 10.3390/plants8110512 31744154 PMC6918357

[B21] Koo D. H. Molin W. T. Saski C. A. Jiang J. Putta K. Jugulam M. (2018). Extrachromosomal circular DNA-based amplification and transmission of herbicide resistance in crop weed *Amaranthus palmeri*. *PNAS* 115 3332–3337. 10.1073/pnas.1719354115 29531028 PMC5879691

[B22] Kreuz K. Fonné-Pfister R. (1992). Herbicide-insecticide interaction in maize: Malathion inhibits cytochrome P450-dependent primisulfuron metabolism. *Pestic. Biochem. Phys.* 43 232–240. 10.1016/0048-3575(92)90036-Y

[B23] Kumar V. Liu R. Boyer G. Stahlman P. W. (2019). Confirmation of 2,4-D resistance and identification of multiple resistance in a Kansas Palmer amaranth (*Amaranthus palmeri*) population. *Pest Manag. Sci.* 75 2925–2933. 10.1002/ps.5400 30843341

[B24] Küpper A. Borgato E. A. Patterson E. L. Goncalves Netto A. Nicolai M. Carvalho S. J. P. (2017). Multiple resistance to glyphosate and acetolactate synthase inhibitors in Palmer amaranth (*Amaranthus palmeri*) identified in Brazil. *Weed Sci.* 65 317–326. 10.1017/wsc.2017.1

[B25] Lu H. Yu Q. Han H. Owen M. J. Powles S. B. (2019). Metribuzin resistance in a wild radish (*Raphanus raphanistrum*) population *via* both *psbA* gene mutation and enhanced metabolism. *Agric. Food Chem.* 67 1353–1359. 10.1021/acs.jafc.8b05974 30640451

[B26] Ma H. Lu H. Han H. Yu Q. Powles S. B. (2020). Metribuzin resistance *via* enhanced metabolism in a multiple herbicide resistant *Lolium rigidum* population. *Pest Manag. Sci.* 76 3785–3791. 10.1002/ps.5929 32452149

[B27] Ma R. Kaundun S. S. Tranel P. J. Riggins C. W. McGinness D. L. Hager A. G. (2013). Distinct detoxification mechanisms confer resistance to mesotrione and atrazine in a population of waterhemp. *Plant Physiol.* 163 368–377. 10.1104/pp.113.223156 23872617 PMC3762656

[B28] Massinga R. A. Currie R. S. Horak M. J. Boyer J. (2001). Interference of Palmer amaranth in corn. *Weed Sci.* 49 202–208. 10.1614/0043-1745(2001)049[0202:iopaic]2.0.co;2

[B29] Mengistu L. W. Christoffers M. J. Lym R. G. (2005). A *psbA* mutation in *Kochia scoparia* (L) Schrad from railroad rights-of-way with resistance to diuron, tebuthiuron and metribuzin. *Pest Manag. Sci.* 61 1035–1042. 10.1002/ps.s1079 15952238

[B30] Molin W. T. Wright A. A. VanGessel M. J. McCloskey W. B. Jugulam M. Hoagland R. E. (2017). Survey of the genomic landscape surrounding the *EPSPS* gene in glyphosate-resistant *Amaranthus palmeri* from geographically distant populations in the United States. *Pest Manag. Sci.* 74 1109–1117. 10.1002/ps.4659 28686355

[B31] Molin W. T. Yaguchi A. Blenner M. A. Saski C. A. (2020). The EccDNA replicon: A heritable, extranuclear vehicle that enables gene amplification and glyphosate resistance in *Amaranthus palmeri*. *Plant Cell* 32 2132–2140. 10.1105/tpc.20.00099 32327538 PMC7346551

[B32] Moore J. W. Murray D. S. Westerman R. B. (2004). Palmer amaranth (*Amaranthus palmeri*) effects on the harvest and yield of grain sorghum (*Sorghum bicolor*). *Weed Technol.* 18 23–29. 10.1614/WT-02-086

[B33] Morgan G. D. Baumann P. A. Chandler J. M. (2001). Competitive impact of Palmer amaranth (*Amaranthus palmeri*) on cotton (*Gossypium hirsutum*) development and yield. *Weed Technol.* 15 408–412. 10.1614/0890-037x(2001)015[0408:ciopaa]2.0.co;2

[B34] Nakka S. Godar A. S. Thompson C. R. Peteron D. E. Juglam M. (2017a). Rapid detoxification via Glutathione S-transferase (GST)-conjugation confers high level of atrazine resistance in Palmer amaranth (*Amaranthus palmeri*). *Pest Manag. Sci.* 73 2236–2243. 10.1002/ps.4615 28500680

[B35] Nakka S. Godar A. S. Wani P. S. Thompson C. R. Peterson D. E. Roelofs J. (2017b). Physiological and molecular characterization of hydroxyphenylpyruvate dioxygenase (HPPD)-inhibitor resistance in Palmer amaranth (*Amaranthus palmeri* S.Wats.). *Front. Plant. Sci.* 8:555. 10.3389/fpls.2017.00555 28443128 PMC5387043

[B36] Nakka S. Thompson C. R. Peterson D. E. Jugulam M. (2017c). Target site-based and non-target site based resistance to ALS inhibitors in Palmer amaranth (*Amaranthus palmeri)*. *Weed Sci.* 65 681–689. 10.1017/wsc.2017.43

[B37] Nandula V. K. Riechers D. E. Ferhatoglu Y. Barrett M. Duke S. O. Dayan F. E. (2019). Herbicide metabolism: Crop selectivity, bioactivation, weed resistance, and regulation. *Weed Sci.* 67 149–175. 10.1017/wsc.2018.88

[B38] Norsworthy J. Ward S. Shaw D. Llewellyn R. Nichols R. Webster T. (2012). Reducing the risks of herbicide resistance: best management practices and recommendations. *Weed Sci.* 60 31–62. 10.1614/WS-D-11-00155.1

[B39] Obenland O. A. Ma R. O’Brien S. R. Lygin A. V. Riechers D. E. (2019). Carfentrazone-ethyl resistance in an *Amaranthus tuberculatus* population is not mediated by amino acid alterations in the PPO2 protein. *PLoS One* 14:e0215431. 10.1371/journal.pone.0215431 30986256 PMC6464220

[B40] Oliveira M. C. Gaines T. A. Dayan F. E. Patterson E. L. Jhala A. J. Knezevic S. Z. (2018). Reversing resistance to tembotrione in an *Amaranthus tuberculatus* (var. *rudis*) population from nebraska, USA with cytochrome P450 inhibitors. *Pest Manag Sci.* 74 2296–2305. 10.1002/ps.4697 28799707

[B41] Palma-Bautista C. Torra J. Garcia M. J. Bracamonte E. Rojano-Delgado A. M. la Cruz R. A. (2019). Reduced absorption and impaired translocation endows glyphosate resistance in *Amaranthus palmeri* harvested in glyphosate-resistant soybean from Argentina. *J. Agric. Food Chem.* 67 1052–1060. 10.1021/acs.jafc.8b06105 30624921

[B42] Pandian B. A. Sathishraj R. Djanaguiraman M. Vara Prasad P. V. Jugulam M. (2020). Role of cytochrome P450 enzymes in plant stress response. *Antioxidants* 9:454. 10.3390/antiox9050454 PMC7278705 32466087

[B43] Pfaffl M. W. (2001). A new mathematical model for relative quantification in real-time RT-PCR. *Nucl. Acids Res.* 29 2002–2007. 10.1093/nar/29.9.e45 11328886 PMC55695

[B44] Preston C. Tardif F. J. Powles S. B. (1996). “Multiple mechanisms and multiple herbicide resistance in *Lolium rigidum*,” in *Molecular Genetics and Evolution of Pesticide Resistance in Molecular Genetics and Evolution of Pesticide Resistance*, ed. Brown T. M. (Washington, DC: American Chemical Society), 117–129. 10.1007/978-94-011-5538-0_12

[B45] Rangani G. Salas-Perez R. A. Aponte R. A. Knapp M. Craig I. R. Mietzner T. (2019). A novel single-site mutation in the catalytic domain of protoporphyrinogen oxidase IX (PPO) confers resistance to PPO-inhibiting herbicides. *Front. Plant Sci.* 10:568. 10.3389/fpls.2019.00568 31156659 PMC6530635

[B46] Ricci G. Maria F. D. Antonini G. Turella P. Bullo A. Stella L. (2005). 7-Nitro-2,1,3-benzoxadiazole derivatives, a new class of suicideinhibitors for glutathione S-transferases. Mechanism of action of potential anticancer drugs. *J. Biol. Chem.* 280 26397–26405. 10.1074/jbc.M503295200 15888444

[B47] Salas R. A. Burgos N. R. Tranel P. J. Singh S. Glasgow L. Scott R. C. (2016). Resistance to PPO-inhibiting herbicide in palmer amaranth from arkansas. *Pest Manag. Sci.* 72 864–869. 10.1002/ps.4241 26817647 PMC5069602

[B48] Salas-Perez R. A. Burgos N. R. Rangani G. Singh S. Refatti J. P. Piveta L. (2017). Frequency of Gly-210 deletion mutation among protoporphyrinogen oxidase inhibitor-resistant Palmer amaranth (*Amaranthus palmeri*) populations. *Weed Sci.* 65 718–731. 10.1017/wsc.2017.41

[B49] Sellers B. A. Smeda R. J. Johnson W. G. Kendig J. A. Ellersieck M. R. (2003). Comparative growth of six *Amaranthus* species in Missouri. *Weed Sci.* 51 329–333. 10.1614/0043-1745(2003)051[0329:cgosas]2.0.co;2

[B50] Shergill L. S. Bish M. D. Jugulam M. Bradley K. W. (2018). Molecular and physiological characterization of six-way resistance in an *Amaranthus tuberculatus* var. *rudis* biotype from Missouri. *Pest Manag. Sci.* 74 2688–2698. 10.1002/ps.5082 29797476

[B51] Singh S. Singh V. Lawton-Rauh A. Bagavathiannan M. V. Roma-Burgos N. (2018). EPSPS gene amplification primarily confers glyphosate resistance among Arkansas Palmer amaranth (*Amaranthus palmeri*) populations. *Weed Sci.* 66 293–300. 10.1017/wsc.2017.83

[B52] Singh S. Singh V. Salas-Perez R. A. Bagavathiannan M. V. Lawton-Rauh A. Roma-Burgos N. (2019). Target-site mutation accumulation among ALS inhibitor-resistant Palmer amaranth. *Pest Manag. Sci.* 75 1131–1139. 10.1002/ps.5232 30298618

[B53] Sosnoskie L. M. Webster T. M. Kichler J. M. MacRae A. W. Grey T. L. Culpepper A. S. (2012). Pollen-mediated dispersal of glyphosate-resistance in Palmer amaranth under field conditions. *Weed Sci.* 60 366–373. 10.1614/WS-D-11-00151.1

[B54] Tranel P. J. Wright T. R. (2002). Resistance of weeds to ALS-inhibiting herbicides: What have we learned? *Weed Sci.* 50 700–712. 10.1614/0043-1745(2002)050[0700:rrowta]2.0.co;2

[B55] Van Wychen L. (2019). *Survey of the Most Common and Troublesome Weeds in Broadleaf Crops, Fruits & Vegetables in the United States and Canada. Weed Science Society of America National Weed Survey Dataset.* Available online at: http://wssa.net/wp-content/uploads/2019-Weed-Survey_broadleaf-crops.xlsx

[B56] Varanasi V. K. Brabham C. Norsworthy J. K. (2018). Confirmation and characterization of non–target site resistance to fomesafen in Palmer amaranth (*Amaranthus palmeri*). *Weed Sci.* 66 702–709. 10.1017/wsc.2018.60

[B57] Varanasi V. K. Brabham C. Norsworthy J. K. Nie H. Young B. Houston M. (2017). A statewide survey of PPO-inhibitor resistance and the prevalent target-site mechanisms in Palmer amaranth (*Amaranthus palmeri*) accessions from Arkansas. *Weed Sci.* 66 149–158. 10.1017/wsc.2017.68

[B58] Vila-Aiub M. M. Neve P. Steadman K. J. Powles S. B. (2005). Ecological fitness of a multiple herbicide-resistant *Lolium rigidum* population: dynamics of seed germination and seedling emergence of resistant and susceptible phenotypes. *J. Appl. Ecol.* 42 288–298. 10.1111/j.1365-2664.2005.01017.x

[B59] Whaley C. M. Wilson H. P. Westwood J. H. (2007). A new mutation in plant ALS confers resistance to five classes of ALS-inhibiting herbicides. *Weed Sci.* 55 83–90. 10.1614/WS-06-082

[B60] Yu Q. Powles S. B. (2014). Resistance to AHAS inhibitor herbicides: current understanding. *Pest. Manag. Sci.* 70 1340–1350. 10.1002/ps.3710 24338926

