# Corrigendum: Predominance of Metabolic Resistance in a Six-Way-Resistant Palmer Amaranth (*Amaranthus palmeri*) Population

**DOI:** 10.3389/fpls.2021.676617

**Published:** 2021-03-31

**Authors:** Chandrima Shyam, Ednaldo A. Borgato, Dallas E. Peterson, Johanna Anita Dille, Mithila Jugulam

**Affiliations:** Department of Agronomy, Kansas State University, Manhattan, KS, United States

**Keywords:** metabolism, inhibitor assays, *EPSPS* amplification, cytochrome P450 monooxygenases, glutathione *S*-transferases

There is an error in the Data Availability statement. The correct accession number for ALS and psbA sequences are **MW361337** and **MW361342**.

The authors apologize for this error and state that this does not change the scientific conclusions of the article in any way. The original article has been updated.

